# Antinociceptive, muscle relaxant and sedative activities of gold nanoparticles generated by methanolic extract of *Euphorbia milii*

**DOI:** 10.1186/s12906-015-0691-7

**Published:** 2015-05-29

**Authors:** Nazar Ul Islam, Ibrahim Khan, Abdur Rauf, Naveed Muhammad, Muhammad Shahid, Mohammad Raza Shah

**Affiliations:** Department of Pharmacy, Sarhad University of Science and Information Technology, Peshawar, Pakistan; Institute of Chemical Sciences, University of Peshawar, Peshawar, Pakistan; Department of Pharmacy, Abdul Wali Khan University, Mardan, Pakistan; H.E.J. Research Institute of Chemistry, International Centre for Chemical and Biological Sciences, University of Karachi, Karachi, Pakistan

**Keywords:** Gold nanoparticles, *Euphorbia milii*, Nanotechnology, Herbal drugs, Antinociceptive, Muscle relaxant, Sedative

## Abstract

**Background:**

Nanotechnology has potential future for enhancing therapeutic efficacy and reducing the unwanted effects of herbal drugs. The biological research on *Euphorbia* species has been supported by the use of some plants in traditional medicines. Many species of *Euphorbia* have been reported as having strong sedative and analgesic effects. In the present research work gold nanoparticles of *Euphorbia milii* methanolic extract (Au-EM) were synthesized, characterized and tested for antinociceptive, muscle relaxant and sedative activities.

**Methods:**

Au-EM was prepared by stirring 1 mM warm trihydrated tetrachloroaurate solution with *E. milii* methanolic extract without using any external reducing agents. The gold nanoparticles were characterized by UV-Visible spectroscopy, infrared spectrophotometery, atomic force microscopy and scanning electron microscopy while their stability was evaluated against varying pH and different volumes of sodium chloride (NaCl). The metal sensing capacity of Au-EM was tested towards cobalt, copper, lead, mercury and nickel. Au-EM was evaluated in BALB/c mice at a dose of 10 and 20 mg/kg for antinociceptive, muscle relaxant and sedative activities in comparison with the crude *E. milii* methanolic extract.

**Results:**

Au-EM showed remarkable stability in different NaCl and pH solutions. Au-EM produced significant (*P* < 0.01) antinociceptive effect at doses of 10 and 20 mg/kg as compared to the crude *E. milii* methanolic extract. In the rotarod test, Au-EM showed significant muscle relaxant effect at 10 mg/kg (*P* < 0.05) and 20 mg/kg (*P* < 0.01) after 30, 60 and 90 min. In an open field test significant sedative effect (*P* < 0.05) of Au-EM was observed at 10 and 20 mg/kg. Moreover significant detection sensitivity was demonstrated towards all the tested heavy metals.

**Conclusions:**

These results concluded that the gold nanoparticles improved the potency of *E. milii* methanolic extract and exhibited significant analgesic, muscle relaxant and sedative properties. The significant metals sensing ability and enhanced stability in different NaCl and pH solutions may enable us to explore different formulations of *E. milii* gold nanoparticles for potentially effective and safe nano-herbal therapy.

## Background

Herbal medicines have been widely recognized by physicians and patients for their better therapeutic value and fewer adverse effects as compared to modern medicines [1]. There is an increasing need for multi-component remedies for the treatment of chronic and complicated diseases where drugs with single target action often fail [2]. Herbal medicine has already been well established as a valuable source of effective treatment for various human diseases. The phytochemical profiles of herbal remedies can provide hints for designing, screening and developing novel multi-target therapeutics [3]. The therapeutic value of herbal medicines has been attributed to the synergistic effects of a variety of active components; however, most of these active constituents possess insoluble character leading to lower bioavailability and increased systemic clearance [4]. Due to these factors, herbal medicines require unnecessarily high systemic administration [5]. The incorporation of herbal drugs into nanosized drug delivery systems has many advantages, including the improvement of solubility, enhancement of pharmacological activity and stability, protection from toxicity, sustained delivery and protection from physical and chemical degradation [6]. Drug delivery systems within the nanometer size regime can be developed to alter both pharmacological and therapeutic effects of drug molecules [7]. Phytotherapeutics need a scientific approach to deliver the components in a novel manner to increase bioavailability, which can be achieved by designing nano-drug delivery systems for herbal constituents. Application of nanotechnology to herbal drugs may lead to the development of nano-herbal products which will open a new era of herbal drug discovery [1].

The family Euphorbiaceae consists of about 2000 species. The genus *Euphorbia* is the largest genus in medicinal plant kingdom which is widely distributed in China and Pakistan. Some species of *Euphorbia* have been traditionally used for the treatment of skin diseases, gonorrhea, migraine, intestinal parasites and as wart cures [8]. The genus *Euphorbia* has been studied widely for its antiproliferative [9, 10], cytotoxicity [11, 12], tumor promoting [13], antimicrobial [14, 15], antidiarrheal [16], antidipsogenic [17], molluscicidal [18, 19], urease inhibitory [20], angiotensin converting enzyme inhibitory [17], antipyretic [21, 22] and analgesic [23, 24] activities. The biological research on *Euphorbia* species has been supported by the use of some plants in traditional medicines or revealed the new activities on modern pharmacological levels [25]. The sedative, anxiolytic, analgesic, antipyretic and anti-inflammatory activities have been reported for *E. decipen* [24], *E. resinifera* [26, 27], *E. fischeriana* [26]*, E. royleana* [28]*, E. heterophylla* [29]*, E. hirta* [21] and *E. milii* [30].

*Euphorbia milii* commonly known as the crown of Thorns is used for ornamental purposes and have not been reported in folk therapy in Pakistan; however, in Nepal the latex is used for treating strains [31], while in China it is used for the treatment of hepatitis and abdominal edema [32]. The undiluted latex of *E. milii* was found to be irritant to mammalian eyes and skin [33]. Phytochemical studies of *E. milii* revealed the presence of β-sitosterol, cycloartenol, β-amyrin acetate, lupeol, euphol, flavonoids and triterpenes [34, 32]. Some of their diterpene esters of ingenol are potent skin irritants but in contrast to other closely-related ingenol and phorbol derivatives they have no tumor promoting activity [32]. Milliamines isolated from *E. milii* latex exhibited potent molluscicidal activity [19]. In previous study, the crude methanolic extract of *E. milii* showed significant analgesic activity comparable to that of diclofenac sodium [30].

The present work demonstrates green synthesis of gold nanoparticles using a methanolic extract of *E. milii* (Au-EM) with characterization, metals sensing and evaluation for antinociceptive, muscle relaxant and sedative activities in comparison with the crude *E. milii* methanolic extract.

## Methods

### Chemicals

Tetrachloroaurate trihydrate (HAuCl_4_.3H_2_O) was purchased from Merck. Commercial grade methanol was purchased from Haq Chemicals Limited Peshawar, Pakistan.

### Animals

BALB/c mice of either sex weighing 25–30 gm were purchased from the Pharmacology section of the Department of Pharmacy, University of Peshawar, Pakistan. The animals were maintained in a 12 h light/dark cycle at 22 ± 2 °C. Access to food and water was *ad libitum*. Experiments on animals were performed between 9:00 am and 3:00 pm. The experiment protocols were in compliance with the guidelines set forth by the Ethical Committee of the Department of Pharmacy, University of Peshawar, Pakistan.

### Collection and extraction

*E. milii* was collected from Toormang, Razagram area of district Dir, Khyber Pukhtunkhwa province of Pakistan in the month of February, 2010. The plant was identified by Dr. Abdur Rashid (taxonomist) and a voucher specimen (UOP-545) was deposited in the herbarium of the Department of Botany, University of Peshawar. Ariel parts of *E. milii* were shade dried at room temperature for 15 days and were crushed to produce fine powder. The powdered material (10 gm) was soaked in methanol for 5 days and then subjected to repeated extraction until exhaustion of the plant material. The extracts obtained were then concentrated under reduced pressure below 50 °C using a rotary evaporator (yield 39.6%).

### Synthesis of gold nanoparticles

100 mg of methanolic extract of *E. milii* were taken and dissolved in 100 ml of methanol; any suspended particles were removed by filtration. This solution was treated as a source extract (containing plant extract) and was utilized for subsequent operations. 20 ml of 1 mM gold solution was warmed in a flask and 5 ml of source extract was added to it. The mixture was stirred uniformly for 4 h. After 30 min, the solution started changing color from yellow to violet and at the end violet red color was obtained.

### Characterization of gold nanoparticles

**Fig. 1:**
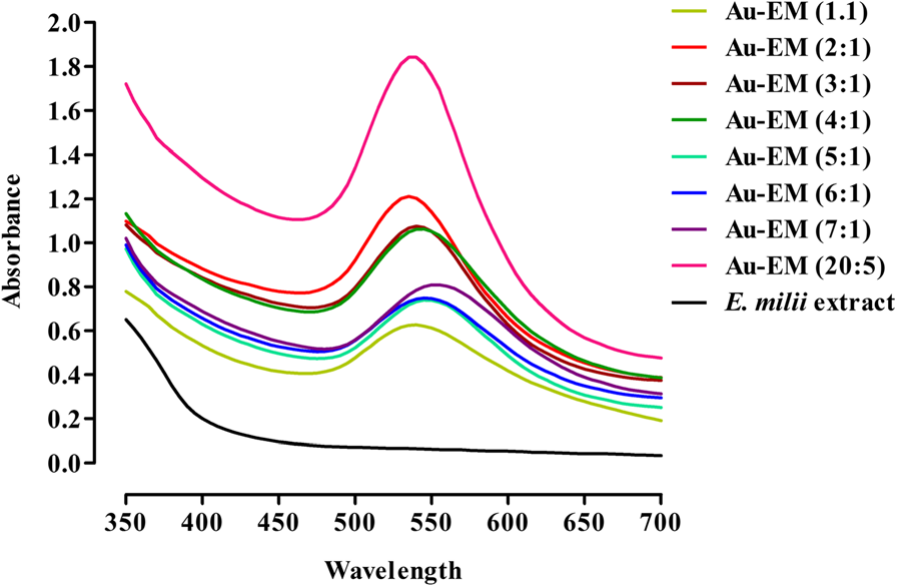
UV-Visible spectra of Au-EM. UV-Visible spectra showing successful formation of Au-EM by observing peaks in the region specified for gold nanoparticles

The gold nanoparticles, which were formed by reacting HAuCl_4_ and *E. milii* were detected by UV-Visible spectrophotometer (Hitachi U-3200, Japan), FTIR (Shimadzu, Prestige-21, Japan), atomic force microscope (Agilent Technologies 5500, USA), scanning electron microscope (JSM*-*5910-JEOL, Japan) and by noticeable color change. By varying the ratio of gold solution and plant extract, the intensity of the peak was changing and a change to visible color was noted. Gold–milii solutions were used in a ratio of 1:1, 2:1, 3:1 and 4:1--- 20:5. The 20 ml 1 mM gold solution and 5 ml plant extract (i.e. 20:5) gave a uniform and sharp peak at 540 nm with an absorbance of 1.89 (Fig. 1). With this ratio a bulk solution was made for further studies.

### Effect of metals ion concentrations

The sensing ability of Au-EM towards different metals was studied at various concentrations of cobalt, copper, lead, mercury and nickel.

### Biological activities

#### Acetic acid induced writhing test

Animals were withdrawn from food 2 hrs before the start of experiment. The animals were divided into four groups. Group I was injected with normal saline (i.p) and served as control while group II was injected with standard diclofenac sodium (10 mg/kg, i.p). The remaining groups were injected (i.p) with 10 and 20 mg/kg of Au-EM. After 30 min of treatment the animals were treated (i.p) with 1 % acetic acid injection. Writhing was counted after 5 min of acetic acid injection and was continued for 10 min [35].

#### Muscle relaxant activity

The rota rod used in this test was a metallic rod (3 cm diameter) coated with rubber and connected to a motor. The rod was rotating at a constant velocity, i.e. 9 rpm and was about 60 cm above the table top in order to prevent the mice from jumping off the roller. Mice were exposed to the rota rod as a pre-test before the experiment and only those mice included in the study that remained on the rod for 5 min at a speed of 9 rpm. All the groups (n = 6) were treated (i.p.) with diazepam (1 mg/kg), saline (10 ml/kg), Au-EM (10 and 20 mg/kg) and *E. milii* extract (50 and 100 mg/kg). Each mouse was allowed for 5 min at the revolving rod and time spent on the rod was recorded [36].

#### Sedative activity

The apparatus used for this activity was consisted of an area of white wood (150-cm diameter) enclosed by stainless steel walls and divided into 19 squares by black lines. The open field was placed in light and sound attenuated room. Animals were acclimatized under red light (40 Watt red bulb) one hour before the commencement of experiment. Animals were administered with saline (10 ml/ kg), diazepam (0.5 mg/kg, i.p), Au-EM (10 and 20 mg/kg, i.p) and *E. milii* extract (50 and 100 mg/kg). After 30 min each animal was placed in the center of box and the numbers of lines crossed were counted for each mouse [37].

### Statistical analysis

The results were expressed as mean ± S.E.M. Significance of difference among mean values were evaluated by one way ANOVA followed by Dunnett’s post test using GraphPad Prism 5 (GraphPad Software Inc. San Diego CA, USA).

## Results

### Characterization of Au-EM

#### UV-Visible spectroscopy

UV-Visible data suggests that the gold nano-sized particles were synthesized successfully by observing peaks with different ratios in the region specified for gold nanoparticles as shown in Fig. 1.

#### Atomic force microscopy and scanning electron microscopy

**Fig. 2:**
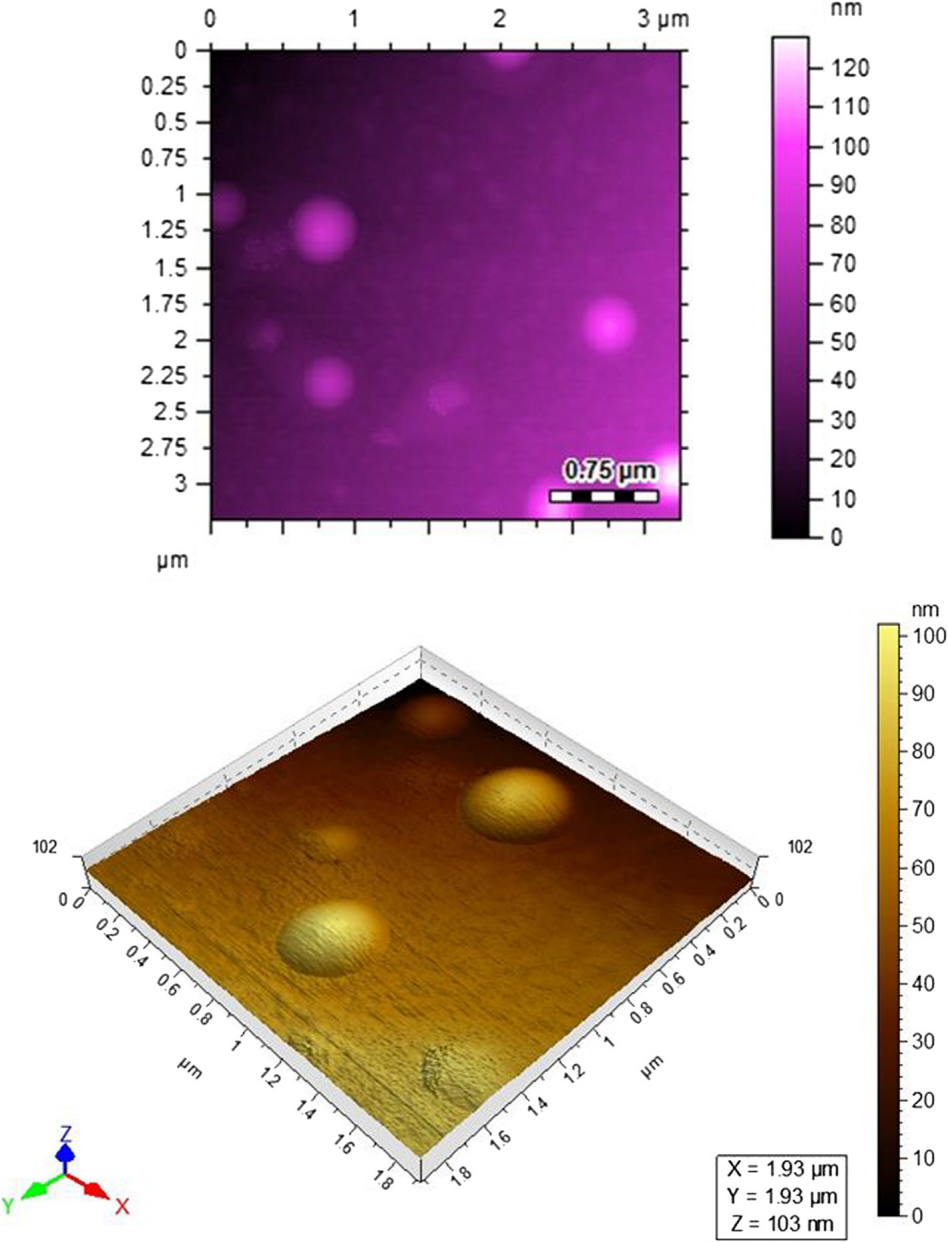
AFM images of Au-EM. Atomic force microscopy images of Au-EM

**Fig. 3:**
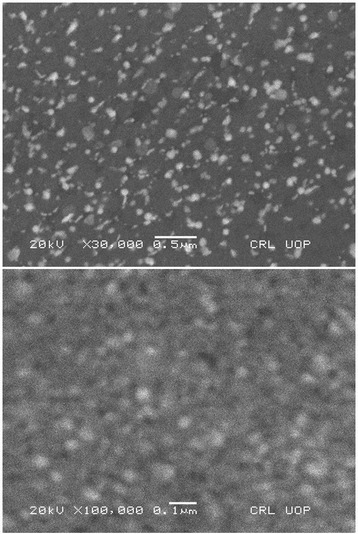
SEM images of Au-EM: Scanning electron microscopy images of *E. milii* gold nanoparticles at different resolutions

Atomic force microscopy (Fig. 2) and scanning electron microscopy (Fig. 3) show that the particle size is in the range of 70–400 nm.

#### Fourier transform infrared spectrophotometry

**Fig. 4:**
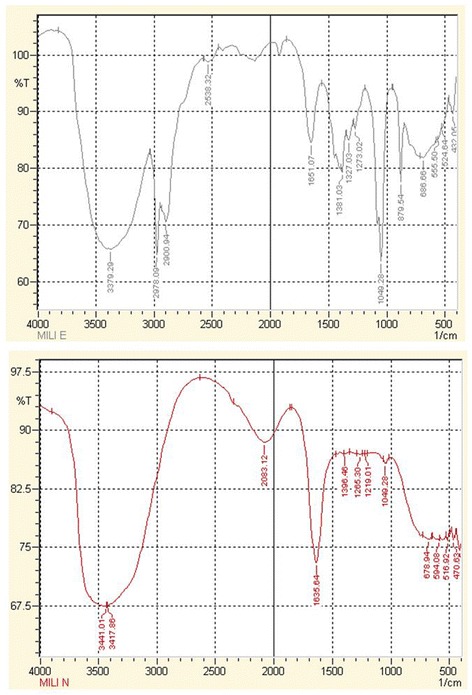
FTIR spectra. Comparison of the FTIR spectra of *E. milii* extract (upper image) and Au-EM (lower image)

Comparing the IR spectra of *E. milii* extract and Au-EM, a broad peak at 3379 cm^−1^ is an indication of NH or OH functionalities present in *E. milii* extract. In case of Au-EM this peak moved towards higher frequency and is broadened more with an increase in intensity, indicating that these functional groups are involved in the reduction of Au^+^ ion to Au^o^ metal. The disappearance of peaks (present in extract) in the region of 2978–2900 cm^-1^ in case of nanoparticles shows the involvement of CH in the stabilization of Au-EM. Similarly shifts in the peaks of *E. milii* extract at 1651 (C = C) and 1381 (C-O) in the spectrum of Au-EM show the reducing and capping ability of these groups (Fig. 4).

### Stability of *E. milli* gold nanoparticles

#### Stability towards pH

**Fig. 5:**
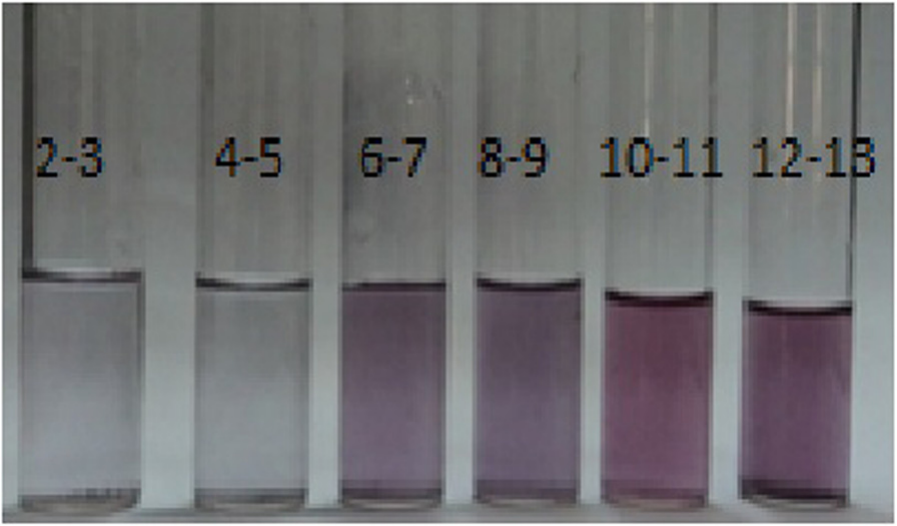
Effect of varying pH on the color of Au-EM. Visible color change after drop wise addition of 1 M HCl or NaOH solutions

**Fig. 6:**
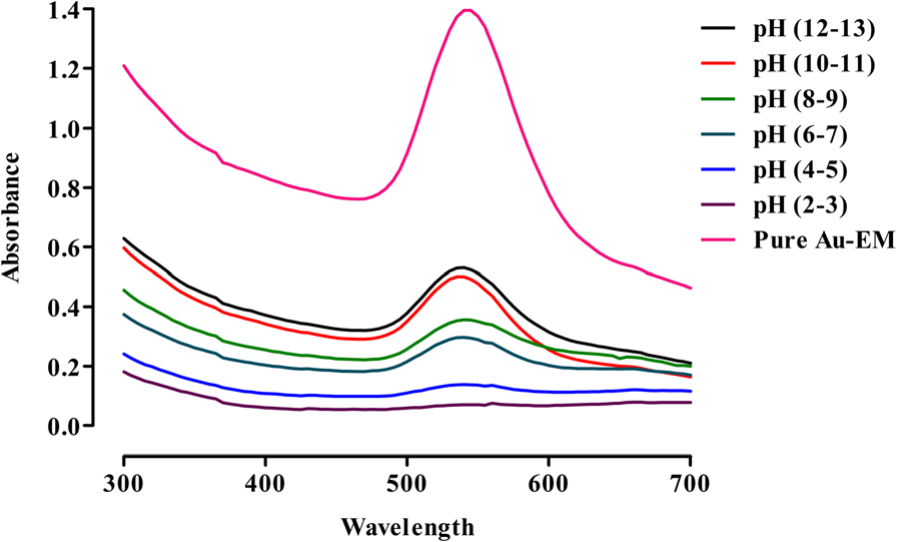
Effect of varying pH on the stability of Au-EM. UV–Vis spectra showing the effect of varying pH on the stability of Au-EM

In order to ascertain the pH effect on the stability of gold nanoparticles of *E. milii,* the pH of the gold nanoparticles solutions were adjusted between 2–14 by drop wise addition of 1 M HCl and NaOH solutions. As shown in Fig. 5, a visible change in color was observed after a change in pH of the solution. This effect was also observed by recording the UV-Visible spectra. It was observed that the gold nanoparticles were stable at alkaline pH and stability started decreasing as the pH was lowered. By gradually lowering the pH from 12 downwards, the number of gold nanoparticles started decreasing and at pH 2–3 it seems that no nanoparticles exist in the solution (Fig. 6).

#### Stability towards sodium chloride

**Fig. 7:**
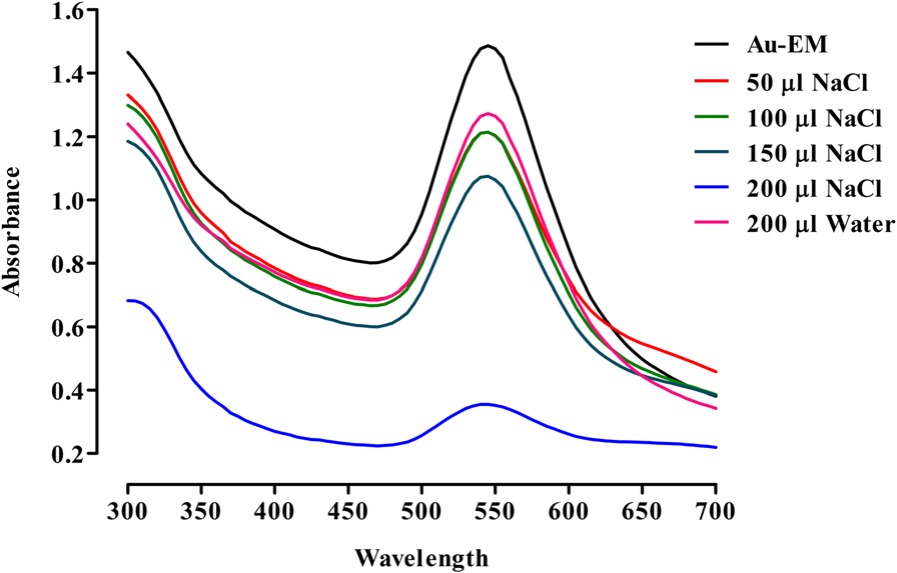
Effect of NaCl on the stability of Au-EM. UV–Vis spectra showing the effect of different volumes of sodium chloride (NaCl) solution on the stability of Au-EM

The effect of salt (NaCl) on the stability of Au-EM was determined by varying the volume of NaCl. It was observed that by increasing the volume of 0.1 M NaCl from 50 μl to 200 μl, the size and number of gold nanoparticles changed. At 200 μl 0.1 M NaCl the peak become broad and its absorbance decreases which indicate that the nanoparticles possess less stability (Fig. 7). Under visible color observation the solution faded from colored to colorless with a final addition forming precipitate.

#### Metals sensing ability of *E. milii* gold nanoparticles

**Fig. 8:**
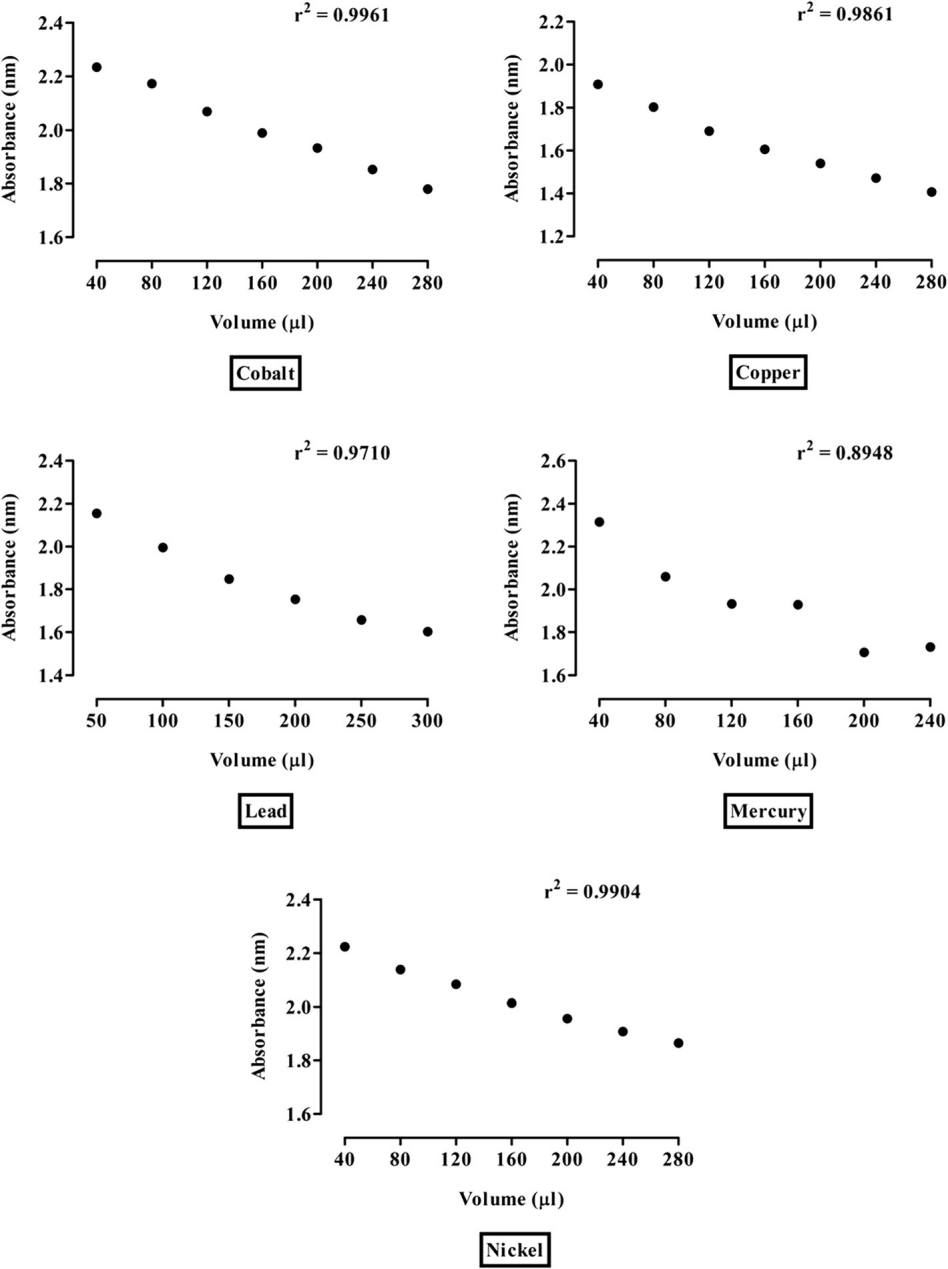
Metals detection sensitivity of Au-EM. The correlation coefficient (r^2^) showing detection sensitivity of Au-EM towards cobalt, copper, lead, mercury and nickel

Au-EM has high detection capacity for all the metals studied. The correlation coefficient demonstrated significant sensitivity for cobalt (r^2^ = 0.9961; *P* < 0.001), copper (r^2^ = 0.9861; *P* < 0.001), lead (r^2^ = 0.9710; *P* < 0.001), mercury (r^2^ = 0.8948; *P* < 0.01) and nickel (r^2^ = 0.9904; *P* < 0.001) as shown in Fig. 8. The r squared value is lower for mercury compared to those for other metals. The removal efficiency increases with increase in the concentration of the metal ions.

### Neuropharmacological activities

#### Antinociceptive activity

**Fig. 9:**
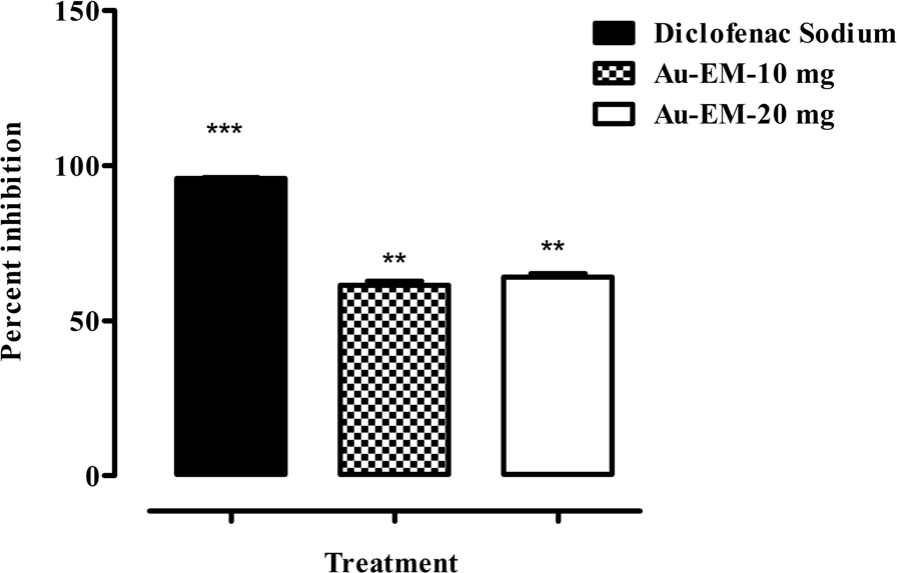
Percent antinociceptive activity of Au-EM. Bars represent the percent inhibition of acetic acid induced pain by Au-EM. **P < 0.01, ***P < 0.001 compared to saline

Significant attenuation (*P* < 0.01) of pain was demonstrated by Au-EM at 10 and 20 mg/kg (Table 1). A dose dependent relief in pain was observed at all the tested doses. Maximum writhing inhibition was observed at a dose of 20 mg/kg (79.86%) as compared to diclofenac sodium (85.21%). The percent inhibition of writhing is shown in Fig. 9.

**Table 1:** Effect of *E. milii* gold nanoparticles (Au-EM) in acetic acid induced writhing test

Treatment	Dose	No. of writhing (10 min)
Saline	10 ml/kg	64.80 ± 1.77
Diclofenac sodium	10 mg/kg	10.40 ± 0.98**
Au-EM	10 mg/kg	29.57 ± 2.97**
	20 mg/kg	19.89 ± 3.98**

Values are expressed as mean ± S.E.M.

**P < 0.01 compared to saline. n = 6

#### Muscle relaxant effect

**Fig. 10:**
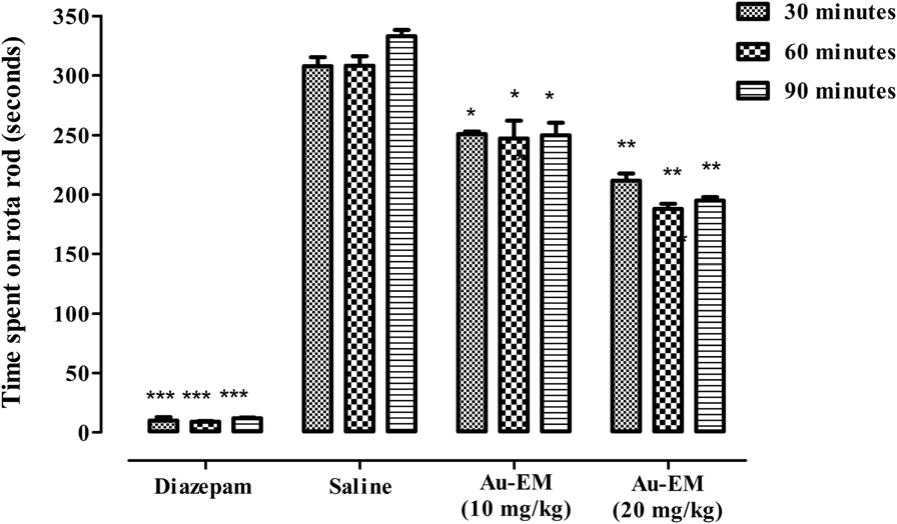
Muscle relaxant activity of Au-EM. Bars represent the time spent in seconds on the rota rod after 30, 60 and 90 min of treatment. *P < 0.05, **P < 0.01, ***P < 0.001 compared to saline

**Fig. 11:**
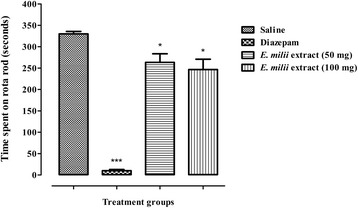
Muscle relaxant activity of *E. milii* methanolic extract. Bars represent the time spent in seconds on the rota rod after 30, 60 and 90 min of treatment. *P < 0.05, ***P < 0.001 compared to saline

The muscle relaxant effect for Au-EM and *E. milii* extract is shown in Figs. 10 and 11 respectively. The time spent on the revolving rod is significantly reduced by Au-EM at 10 mg/kg (*P* < 0.05) and 20 mg/kg (*P* < 0.01) in comparison with the control group. Moreover, significant reduction (*P* < 0.05) in the time was observed with *E. milii* extract at 50 and 100 mg/kg. Diazepam which is a positive control was more significant (*P* < 0.001) than the tested doses of Au-EM and *E. milii* extract.

#### Sedative effect

Au-EM was sedative at all the tested doses. Au-EM as compared to control, cause significant reduction (*P* < 0.05) in the number of lines crossed in 10 min but was found to be less significant as compared to diazepam (*P* < 0.001) (Table 2). No significant effect was observed with *E. milii* extract at the tested doses.

**Table 2:** Open field test of *E. milii* gold nanoparticles (Au-EM)

Treatment	Dose	No of lines crossed in 10 min
Saline	10 ml/kg	126 ± 1.23
Diazepam	0.5 mg/kg	5 ± 0.02***
Au-EM	10 mg/kg	110.67 ± 2.76*
	20 mg/kg	108.98 ± 1.98*
*E. milii* extract	50 mg/kg	125.87 ± 2.65
	100 mg/kg	127.98 ± 2.87

Values represent the number of lines crossed in 30 min

*P < 0.05, ***P < 0.001 compared to saline. n = 6

## Discussion

Nanotechnology has a dramatic impact on medicine [38]. The application of nanotechnology for treatment, diagnosis, monitoring, and control of biological systems has recently been determined as nanomedicine [39]. Nano-sized drug delivery systems of herbal drugs have a potential future for enhancing the activity and overcoming problems associated with the plant medicines [40]. Our study is the first to report the potent antinociceptive, muscle relaxant and sedative activities of gold nanoparticles generated from *E. milii* methanolic extract. Different species of *Euphorbia* have been used in different doses for curing various ailments. *E. thomsoniana* latex is used in doses of 125–250 mg for treating abdominal disorders [41]. *E. prostrata* at a dose of 750 mg relieved the symptoms of bronchial asthma [42] while in a dose of 100 mg once daily for 14 days, it is useful in ameliorating the signs and symptoms associated with hemorrhoids [43, 44]. There are several studies on a possible tumor promoter activity of *E. milii* chemical constituents [45–47]; however, an *in-vivo* study contradicts this activity [32]. Moreover, the tumor induction property of various species of *Euphorbia* is a long-term effect and is not a concern for their short term use after *in-vivo* administration. Gold nanoparticles show several features that make them well suited for biomedical applications including their ease of synthesis, high surface area, stability and low inherent toxicity [48, 49]. In this study, gold nanoparticles of *E. milii* were synthesized without utilizing any external reducing agents. De Matos et al. synthesized silver nanoparticles using *E. milii* latex and showed that the peptides of proteins present in the latex encapsulate the silver nanoparticles thereby preventing aggregation [50]. Valodkar and others synthesized silver nanoparticles using the stem latex of *E. nivulia* and concluded that the major component, Euphol acts as a reducing agent and stabilization was achieved by certain peptides and terpenoids present within the latex [51]. Carbonyl groups of amino acids and peptides of proteins have a strong binding affinity to metals and therefore act as encapsulating agents and thus prevents nanoparticles agglomeration [52]. Phytochemical analysis of *E. milii* methanolic extract showed that the extract is rich in cardiac glycosides, steroids/phytosterols, anthocyanin, terpenoids, flavonoids and tannins [53].

The stability of nanoparticles in aquatic environment plays an important role in determining their environmental implication and potential risk to human health [54]. In this study gold nanoparticles of *E. milii* were found to be stable at alkaline pH and stability started decreasing as the pH was lowered. The reason of instability of gold nanoparticles at lower pH was might be due to the removal of stabilizer (plant extract) from the gold surface therefore destabilizing the Ag^0^ nanoparticles. Lower pH may also caused oxidation of neutral Au^0^ nanoparticles to Au^+^ ions. Furthermore by increasing the volume of 0.1 M NaCl from 50–200 μl, the size and the number of gold particles changed. When exposed to various pH and salt concentrations, the nanoparticles respond with physicochemical changes to their material structure and surface characteristics and can be manifested as swelling, dissociating or surface charge switching, in a manner that favors drug release at the target site over surrounding tissues [55, 56]. Chemically stable metallic nanoparticles have no significant cellular toxicity whereas nanoparticles able to be reduced, oxidized or dissolved are cytotoxic and even genotoxic for cellular organisms [57]. The enhanced stability towards varying pH and different volumes of NaCl will enable us to explore different formulations of *E. milii* gold nanoparticles for potentially effective and safe herbal therapy. Moreover, further studies should be warranted like zeta potential measurements to validate the stability of *E. milii* gold nanoparticles.

Analyte-mediated gold nanoparticle aggregation has been particularly useful in the detection of proteins, DNA, glucose, antibodies, toxic metal ions and other substances [58]. Metal based nanomaterials have been explored to have great potential to remove a variety of heavy metals such as mercury, arsenic, lead, nickel, copper, cadmium and chromium [59]. Heavy metals are highly toxic and possess severe threat to the environment and human health [60]. Heavy metals are a known contaminant or adulterant of many traditional remedies [61]. Herbal medicines contaminated with heavy metals arise from contaminated agricultural lands and/or the production process [62]. Therefore the determination of heavy metal content in herb is important for both toxicological and acceptance criteria. In this study the gold nanoparticles of *E. milii* showed significant detection capacity towards cobalt, copper, lead, mercury and nickel. Application of nanoparticles to the development of a sensitive and simple method for the rapid evaluation of heavy metals levels is highly desirable for herbal monitoring and food safety applications. These nanoparticles have the potential to be used for preventive strategies and may be an effective therapy for heavy metals chelation. Moreover, analytical studies should be performed for the presence of bioactive silica micro- and nanoparticles [63, 64] which are formed in addition to the gold nanoparticles. This may have implication for the safety of nano-herbal products.

Acetic acid-induced writhing test is a well recommended protocol in evaluating medicinal agents for their antinociceptive property. The nociceptive response was due to the production of prostaglandins induced by acetic acid through the action of the constitutive enzyme cyclooxygenase-I and its isoform cyclooxygenase-II [65]. In our previous study, the reduction in the mean number of writhings for the crude methanolic extract were 12.34, 32.54 and 71.44% at the tested doses of 50, 100 and 150 mg/kg respectively [30], however for Au-EM the maximum reduction in the mean number of writings was 79.86% at a dose of 20 mg/kg. The result revealed increased antinociceptive effect of Au-EM at a lower dose relative to the crude methanolic extract. In the muscle coordination test, significant skeletal muscle relaxation was produced at 10 mg/kg (*P* < 0.05) and 20 mg/kg (*P* < 0.01) after 30, 60 and 90 min. Demonstration of marked muscle relaxant effect by the rota-rod study indicated that Au-EM induced neurological deficit accompanied with taming or calming effect in mice, supporting its CNS depressant effect. As compared to Au-EM, the *E. milii* extract alone was found to be less significant (*P* < 0.05) at 50 and 100 mg/kg. Furthermore, no significant effect in the locomotor activity was observed with crude *E. milii* methanolic extract however a significant decrease (*P* < 0.01) was observed with Au-EM as compared to diazepam (*P* < 0.001), thus indicating that its sedative properties is due to its motor depressant effect in mice. Most of the centrally acting analgesics influence the locomotor activities by reducing the motor activity because of their CNS depressant property [66]. Locomotor activity is considered as an index of wakefulness or alertness of mental activity and a decrease activity is indicative of calmness and sedation as a result of reduced CNS excitability [67]. Au-EM treatment significantly influenced the locomotor activity of mice by demonstrating a decrease in the number of lines crossed in 10 min and hence indicating its CNS depressant property. Nanotechnological strategies change a substance’s properties and behavior in a biological environment and can potentiate the actions of plant extracts, promote sustained release of active ingredients, reduced the required dose, decrease the side effects and improve activity [4].

## Conclusions

Gold nanoparticles of *E. milii* methanolic extract were prepared without utilizing any external reducing agents. The nanoparticles showed enhanced stability in different NaCl and pH solutions. Moreover, the gold nanoparticles improved the potency of *E. milii* methanolic extract and exhibited significant analgesic, muscle relaxant and sedative properties. Further behavioral, biochemical and pharmacokinetic studies are required to validate the potency of these nanoparticles which may enable us to formulate *E. milii* as potentially effective and safe nano-herbal therapy.
